# Aged Mouse Hippocampus Exhibits Signs of Chronic Hypoxia and an Impaired HIF-Controlled Response to Acute Hypoxic Exposures

**DOI:** 10.3390/cells11030423

**Published:** 2022-01-26

**Authors:** Brina Snyder, Hua-Kang Wu, Brianna Tillman, Thomas F. Floyd

**Affiliations:** 1 Department of Anesthesiology and Pain Management, University of Texas Southwestern Medical Center, Dallas, TX 75390, USA; Brina.Snyder@UTSouthwestern.edu (B.S.); Hua-kang.Wu@UTSouthwestern.edu (H.-K.W.); Brianna.Tillman@UTSouthwestern.edu (B.T.); 2 Department of Cardiothoracic Surgery, University of Texas Southwestern Medical Center, Dallas, TX 75390, USA

**Keywords:** HIF, hypoxia, age, hippocampus, memory

## Abstract

Altered hypoxia-inducible factor-alpha (HIF-α) activity may have significant consequences in the hippocampus, which mediates declarative memory, has limited vascularization, and is vulnerable to hypoxic insults. Previous studies have reported that neurovascular coupling is reduced in aged brains and that diseases which cause hypoxia increase with age, which may render the hippocampus susceptible to acute hypoxia. Most studies have investigated the actions of HIF-α in aging cortical structures, but few have focused on the role of HIF-α within aged hippocampus. This study tests the hypothesis that aging is associated with impaired hippocampal HIF-α activity. Dorsal hippocampal sections from mice aged 3, 9, 18, and 24 months were probed for the presence of HIF-α isoforms or their associated gene products using immunohistochemistry and fluorescent in situ hybridization (fISH). A subset of each age was exposed to acute hypoxia (8% oxygen) for 3 h to investigate changes in the responsiveness of HIF-α to hypoxia. Basal mean intensity of fluorescently labeled HIF-1α protein increases with age in the hippocampus, whereas HIF-2α intensity only increases in the 24-month group. Acute hypoxic elevation of HIF-1α is lost with aging and is reversed in the 24-month group. fISH reveals that glycolytic genes induced by HIF-1α (lactose dehydrogenase-a, phosphoglycerate kinase 1, and pyruvate dehydrogenase kinase 1) are lower in aged hippocampus than in 3-month hippocampus, and mRNA for monocarboxylate transporter 1, a lactose transporter, increases. These results indicate that lactate, used in neurotransmission, may be limited in aged hippocampus, concurrent with impaired HIF-α response to hypoxic events. Therefore, impaired HIF-α may contribute to age-associated cognitive decline during hypoxic events.

## 1. Introduction

Methods to delay cognitive decline associated with aging and lengthen “health-span” (the period of time an individual lives disease-free) have become a primary concern as the global average life-span continues to increase [1]. Contributors to shortened health-span include chronic diseases (i.e., hypertension, chronic heart failure, chronic obstructive pulmonary disorder, obstructive sleep apnea, anemia, diabetes, etc.) which impair tissue oxygenation, known as hypoxia [2,3,4,5]. While many individuals experience mild impairments in cognitive recall with age [6], others experience progressive declines in recognition, temporal, and/or procedural memory. The mechanisms underlying the various types of cognitive aging an individual will experience are complex, but it is interesting to note that aging-related diseases associated with hypoxia accelerate cognitive impairment [5,7,8,9,10,11,12,13,14].

Disease-induced hypoxia may hinder adaptive responses to acute hypoxic exposures [15,16]. Acute hypoxia is episodic yet frequently experienced over the course of a lifetime, especially during periods of disease exacerbation, physical exertion, and travel to higher elevations; in inflammatory responses; and during or in recovery from surgical interventions, to name a few. Evidence also suggests that aged individuals have less cardiac, pulmonary, and cerebrovascular reserve to accommodate acute hypoxic events [17,18]. Impaired vascular reactivity is evident in aged cerebral tissue and is associated with cognitive impairment [5,19,20].

Diminished neurovascular coupling has been observed in aged hippocampal formation [19,20,21,22,23], which is necessary for the storage and retrieval of working and temporal memory [24,25,26,27]. The hippocampus is less vascularized than other cortical areas [19] and particularly susceptible to hypoxia, even in young adult models [13,21,23,28,29,30]. Delayed oxygen delivery, or the inability to efficiently adapt to hypoxia, may thus underlie slower memory recall observed in aging, and chronic hypoxia due to age-related diseases may dysregulate protective molecular responses, predisposing those individuals to the onset of dementias following acute hypoxic events [5].

**Figure 1:**
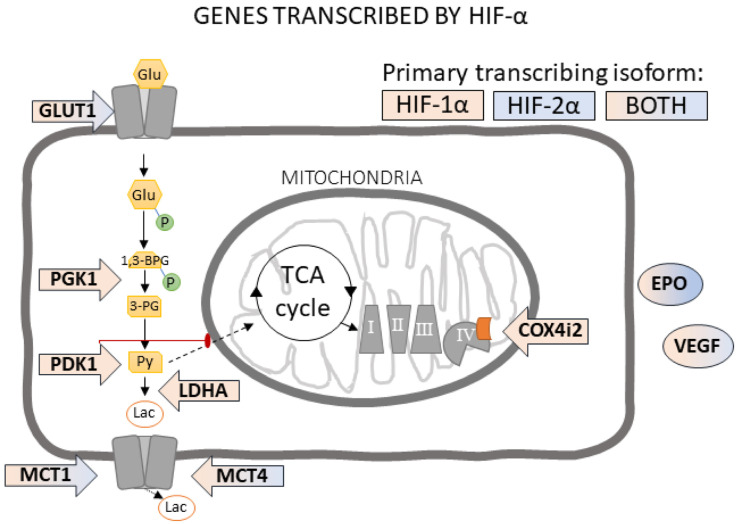
Genes of interest transcribed by HIF-α isoforms. HIF-1α transcribes metabolic switching genes lactose dehydrogenase- A (*LDHA*), phosphoglycerate kinase 1 (*PGK1*), pyruvate dehydrogenase kinase 1 (*PDK1*), and switched cyclooxygenase 4 (*COX4*) from isoform 1 to isoform 2. Both HIF-1α and HIF-2α have been reported to transcribe glucose transporter 1 (*GLUT1*), monocarboxylate transporters (*MCT*) 1 and 4, erythropoietin (*EPO*), and vascular endothelial growth factor (*VEGF*) in a cell-specific manner. HIF-2α is particularly involved in modulating the EPO signal. Glu = glucose; 1,3-BPG = 1,3-biphosphoglycerate; 3-PG = 3-phosphoglycerate; Py = pyruvate; Lac = lactate; TCA = tricarboxylic acid cycle; I, II, III, and IV = complexes I, II, III, and IV of electron transport chain.

Hypoxia-inducible factor-alpha (HIF-α) is present in at least three known isoforms (HIF-1α, HIF-2α, and HIF-3α) and serves as the master transcription regulator of cellular hypoxic responses to increase vascularization, temporarily switch from oxidative phosphorylation to glycolysis, and modulate reactive oxygen species and the cell cycle to promote survival [31]. There is some transcriptional overlap between HIF-α isoforms, as observed in the transcription of vascular endothelial growth factor (*Vegfa*), glucose transporter 1 (*Slc2a1*), and monocarboxylate transporters (*Slc16a1* & *Slc16a4*) [32]. However, many tissue beds and cell types exhibit preferential roles for each isoform. For instance, cortical neurons primarily express HIF-1α, while cortical astrocytes primarily express HIF-2α [33]. HIF-1α primarily mediates genes which switch energetics from oxidative phosphorylation to glycolysis (e.g., lactose dehydrogenase-A [*Ldha*], phosphoglycerate kinase 1 [*Pgk1*], pyruvate dehydrogenase kinase 1 [*Pdk1*], and cyclooxygenase 4 isoform 2 [*Cox4i2*]) [32], whereas the expression of erythropoietin (*Epo)* and cell growth genes are under the transcriptional control of HIF-2α in the cells where it is expressed [34,35,36,37] (Figure 1). Further, HIF-1α predominantly responds to acute hypoxia, while HIF-2α is active under chronic and mild hypoxic exposures [38,39]. Because of its high impact on cell survival and metabolism, HIF-α is highly regulated and degraded at normal oxygenation [40,41] by prolyl hydroxylases (PHD), von Hippel Lindau protein (VHL), and factor inhibiting HIF-1 (FIH).

HIF-1α and HIF-2α have both been reported in the hippocampus, and their gene products appear to enhance cognitive function and neuronal signaling. For example, the protein EPO acts as a neuromodulator and enhances spatial memory following either hypoxia or hippocampal insults through activation of post-synaptic JAK-STAT pathways and the release of BDNF [42,43,44,45]. Lactate is a major substrate for synaptic plasticity [46,47], although to what degree its expression in the hippocampus relies on HIF-1α has not been explored. Inhibition of prolyl-hydroxylases (PHD), which target HIF-α to degradation, also improves cognitive outcomes, suggesting that HIF-α is necessary for maintaining memory under hypoxia [42]. Previous studies report that the partial pressure of oxygen is lower in aged cortices than in young cortices and is less responsive to hypoxic exposure [48,49]. Further, HIF-α expression is deficient in aging cortex, and the loss is associated with cognitive impairment [23,50,51,52,53,54,55]. Few studies have focused solely on the function of HIF-α in aged hippocampus [56,57], and even fewer have been focused on mouse hippocampus. This study investigates the hypothesis that aging is associated with impaired hippocampal HIF-α activity.

**Figure 2:**
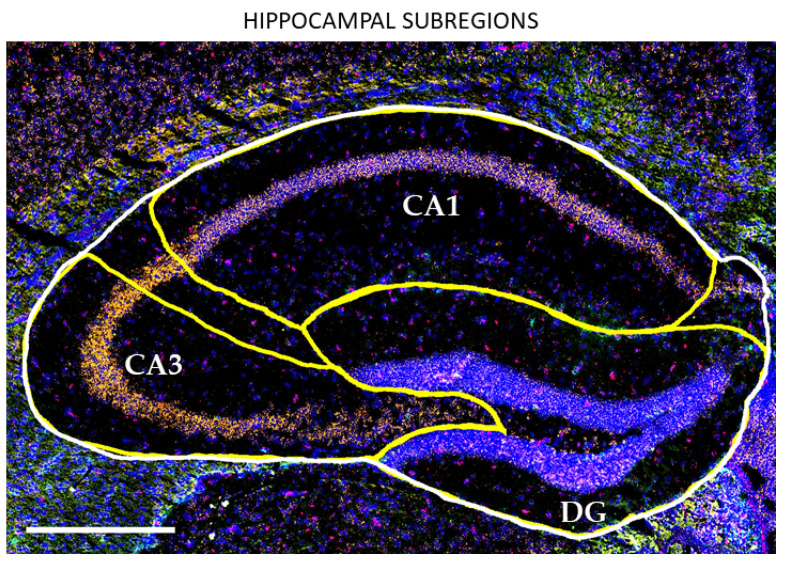
Hippocampal subregions involved in memory acquisition. Representative in situ hybridization image of the hippocampus outlining the regions of the hippocampus which are integral to declarative memory. The dentate gyrus (DG) and CA3 subregions receive cortical input from the entorhinal cortex (ETC—not shown) and then synapse onto the CA1. The CA1 integrates inputs an then processes information through the subiculum and ETC for storage. Scale bar = 500 μm; in situ hybridization of mRNA: blue = DAPI; orange = *Ldha*; red = *Glut1*; green = *Gfap*.

Because the dorsal hippocampus is integral to declarative memory [26], dysregulation of HIF-α within the hippocampus may be of particular concern in the context of cognitive aging, especially with chronic and/or acute hypoxia. Hippocampal subregions (dentate gyrus [DG], CA3, and CA1; Figure 2) receive input from neocortical areas through the perforant and temporoammonic pathways originating from layers 2 and 3 of the entorhinal cortex (ETC). Mossy fibers from the DG synapse onto pyramidal cells of the CA3, which in turn synapse onto dendrites of CA1 pyramidal cells through Schaffer collaterals. CA1 pyramidal cells pass information through the subiculum back to the ETC, allowing for the integration and storage of the multiple data inputs involved in declarative memory [28,29,58]. Thus, this study also investigates the spatial expression of HIF-1α and HIF-2α and their gene products to determine whether one or more subregions of the hippocampus are more or less impacted by changes in HIF-α activity during aging.

## 2. Materials and Methods

### 2.1. Animal Care

Animal work described in this study has been approved and conducted under the oversight of the UT Southwestern Institutional Animal Care and Use Committee. UT Southwestern uses the “Guide for the Care and Use of Laboratory Animals” when establishing animal research standards. Male and female C57Bl/6j mice 12 weeks (3 mo), 40-week (9 mo), and 77 weeks (18 mo) old were purchased from Jackson Laboratories (Bar Harbor, ME, USA). Twenty-four-month (24 mo)-old male and female B6.Cg-Tg(Thy1-YFP)HJrs/J mice were a gift from Drs. Ann Stowe (University of Kentucky, Lexington, KY, USA) and Mark Goldberg (UT Health Science Center, San Antonio, TX, USA) and are available from Jackson Laboratories (Stock No: 003782) [59]. These mice exhibit YFP in sensory and motor neurons, layer IV of cortical neurons, and a subset of hippocampal pyramidal neurons with no reported phenotypic, toxic, or synaptic effects due to YFP expression [59,60].

Based on a power analysis using effect sizes determined from preliminary fISH data acquired in our laboratory (power of 0.80 and alpha of 0.05), 5–6 animals were included in each group (G*Power, v. 3.1.9.4, Universität Kiel, Kiel, Germany). All mice were group-housed (3–4/cage) in temperature-controlled rooms under a 12:12 h light:dark cycle. Upon arrival in the animal facility, animal numbers were coded and randomized to a treatment group using a random sequence generator (random.org). Post-treatment sample preparation and analysis were performed by a technician blinded to the treatment groups. Results were uncoded following statistical analysis.

### 2.2. Acute Hypoxia

One week prior to hypoxia treatments, home cages were transferred into A-Chambers connected to an A84XOV controller (Biospherix, Ltd., Parish, NY, USA) for acclimation to the environment. On the day of treatment, oxygen within the chambers was set to either 21% (normoxic) or 8% O_2_ (balance nitrogen; hypoxic) for 3 h. Immediately after exposure, mice were deeply anesthetized with isoflurane under the corresponding treatment oxygen concentration, followed by decapitation. Brains were harvested as described below.

### 2.3. Tissue Processing

Brains were rapidly rinsed and cooled in ice-cold HBSS and then placed into an ice-cold coronal matrix. Coronal sections 3 mm thick containing the dorsal hippocampus were collected and embedded in Tissue Tek OCT (Sakura Finetek, Torrance, CA, USA), flash frozen in 2-methyl-butane, then stored at −80 °C until processed and mounted onto slides.

### 2.4. Slide Preparation 

Frozen tissue samples were randomly selected by an experimenter blinded to age and treatment for cryosectioning. Tissue was equilibrated to −20 °C within a cryostat (Leica CM3050 S, Leica, Buffalo Grove, IL, USA). Two 10 µm coronal sections containing the dorsal hippocampus (Bregma −1.43 to −2.69, Paxinos and Franklin, 5th ed.) were then serially mounted onto each slide 250 μm apart. The slides were stored at −80 °C in sealed slide boxes for future applications. Three slides spanning the collected area were randomly selected to confirm orientation and quality.

### 2.5. Fluorescent In Situ Hybridization (fISH)

Two sections per animal were selected for analysis of mRNA expression of each gene of interest by fISH. fISH was performed using a prepared kit (Quantigene ViewRNA ISH Cell Assay, Affymetrix, Santa Clara, CA, USA), and associated mRNA probes targeting the genes of interest (*Hif1a*, *Epas1*, *Ldha*, *Slc2a1*, *Pgk1*, *Pdk1*, *Cox4i1*, *Cox4i2*, *Epo*, *EpoR*, *Vegfa*, *Slc16a1, Slc16a4*, *Actb*, and *Gapdh*) were obtained from Thermo Fisher (Carlsbad, CA, USA). Slides were removed from −80 °C and immediately fixed for 30 min at RT in 4% paraformaldehyde, then serially dehydrated in 50%, 70%, and 100% EtOH and stored at −20 °C for subsequent analysis. Within one week of dehydration, slides were rehydrated and prepared for probe incubation according to kit manufacturer’s instructions for samples adhered to coverslips with the following modifications for tissue: rehydration and washing steps were 5 min each, followed by drawing a hydrophobic barrier around each sample. An amount of 120 μL detergent solution was applied directly to each sample for 5 min, followed by washing in PBS by agitation. Probe sets were prepared at 3× the suggested concentration and incubated overnight at 40 °C. Subsequent amplification steps were prepared at a dilution of 1:20 and allowed to incubate 45 min each. Nuclei were labeled with DAPI, and coverslips were applied using Prolong Antifade Mountant (ThermoFisher Scientific, Grand Island, NY, USA). Coverslips were set overnight prior to scanning in a Zeiss Axioscan using Zen 3.2, courtesy of the Whole Brain Microscopy Facility at UTSW. Individual and merged channels from fISH images were thresholded and exported from ZEN Blue 3.1 (Zeiss, White Plains, NY, USA) for analysis in FIJI (ImageJ 1.53c [58]). A technician blinded to groups manually drew regions of interest (ROIs) as the right and left hippocampi and subregions (DG, CA1, and CA3), for each section referencing the coronal sections of the Allen Mouse Brain Atlas(Seattle, WA, USA) [61]. Spot segmentation and quantification in FIJI was scripted to automatically yield the number of spots per area within each ROI for each analyte channel exported.

### 2.6. Immunohistochemistry (IHC) 

One slide immediately before and one slide immediately after the groups of slides selected for fISH analysis were chosen to evaluate protein expression of HIF-1α or HIF-2α within the hippocampus. Each sample was incubated with primary antibodies directly conjugated to their corresponding fluorophore, targeting either HIF-1α-dy650 (1:200, NB100-479C) or HIF-2α-AF647 (1:100, NB100-122AF647; Novus Biologicals, Centennial, CO, USA). Slides were removed from −80 °C storage and immediately fixed in 4% paraformaldehyde for 30 min, followed by washing 3× in PBS and drawing of hydrophobic barriers around each sample. Samples were then permeabilized for 5 min in 0.5% Triton X-100 followed by washing in TBST and then blocking with 2% BSA in TBST for 30 min at RT. Primary antibody cocktails were prepared at the aforementioned dilutions in incubation buffer (1% BSA, 0.3% Triton X-100, remainder TBST), and 100 μL was applied to each sample to incubate overnight at 4 °C. Slides were then washed, nuclei labeled with 1× DAPI (Sigma-Aldrich, St. Louis, MO, USA) for 5 min, then washed again and coverslipped. Fluorescent intensity of each channel was serially acquired on a Leica DMI8 Synapse Thunder system (Leica, Buffalo Grove, IL, USA) with Instant Computational Clearing to reduce background noise. All segmentation and quantification steps were performed in FIJI. ROIs were drawn as outlined above, and the mean pixel intensity per ROI was quantified. 

### 2.7. Statistical Analysis

Statistical analysis was performed using JMP (JMP v. 16.1.0, 2021, SAS Institute, Inc., Cary, NC, USA). Sample identifiers were uncoded following analysis. Two-way ANOVA was used to test for significant effects of age (3 mo normoxic vs. all other ages normoxic) and acute hyxpoxic exposure and interactions, followed by Bonferroni post-hoc analysis. Statistical significance was set at *p* ≤ 0.05. Results are shown as mean ± SEM, unless otherwise indicated.

## 3. Results

### 3.1. HIF-α Levels under Normoxia Are Increased in the Aged Hippocampus

**Figure 3:**
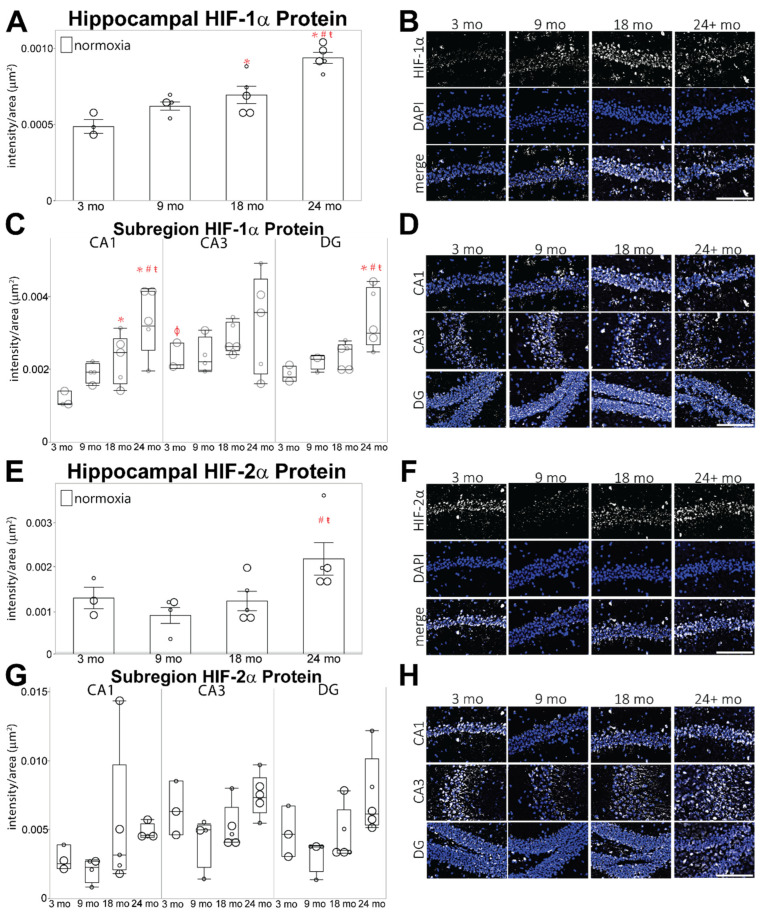
Hippocampal HIF-α protein levels are elevated with age. (**A**,**E**) HIF-1α (**A**) and HIF-2α (**E**) protein reported as intensity/μm^2^ using IHC on hippocampal slices from 3 mo, 9 mo, 18 mo, and 24 mo age groups. (**B**,**F**) Representative images of HIF-1α (**B**) and HIF-2α (**F**) intensity IHC observations. White spots are HIF-1α, and blue spots are DAPI. (**C**,**G**) HIF-1α (**C**) and HIF-2α (**G**) protein measured as intensity/μm^2^ within hippocampal subregions (dentate gyrus [DG], CA1, and CA3) of different aged mice. (**D**,**H**) Representative images of HIF-1α (**D**) and HIF-2α (**H**) intensity within each subregion. Images are displayed as merged channels with HIF-1α or HIF-2α in white and DAPI in blue. Scale bar = 150 μm; *p* ≤ 0.05; * = compared to 3 mo normoxic control; # = compared to 9 mo; ŧ = compared to 18 mo; ϕ = compared to other age-matched regions; small dots = female mice; large dots = male mice.

A main effect of age is observed in the baseline levels of HIF-1α protein within the hippocampus (F = 17.0264, *p* ≤ 0.05; Figure 3A,B). When compared to 3 mo animals, mean HIF-1α intensity is slightly elevated in the total hippocampus by 9 mo and is significantly higher than 3 mo HIF-1α intensity in 18 mo hippocampus. By 24 mo of age, hippocampal HIF-1α is significantly higher than in all other age groups. The pattern of HIF-1α intensity is heterogenous within subregions and changes with age (Figure 3C,D). At 3 mo of age, the CA3 subregion exhibits higher HIF-1α intensity than all other regions. Mean HIF-1α intensity increases throughout aging across all subregions, but the difference between regions is eventually abolished such that all three subregions exhibit equal levels of HIF-1α expression by 24 mo.

Compared to 3-month-old animals, significant increases in the mean intensity of the HIF-2α isoform were only observed in the 24 mo group (F = 5.2533, *p* ≤ 0.05; Figure 3E,F). Similar to HIF-1α, HIF-2α protein levels are higher in the CA3 subregion of the hippocampus, and the intensity pattern across regions changes throughout aging, although these changes are not significantly different from 3 mo HIF-2α intensities in each subregion (Figure 3G,H).

### 3.2. HIF-α Levels under Acute Hypoxia Are Diminished in Aged Hippocampus

**Figure 4:**
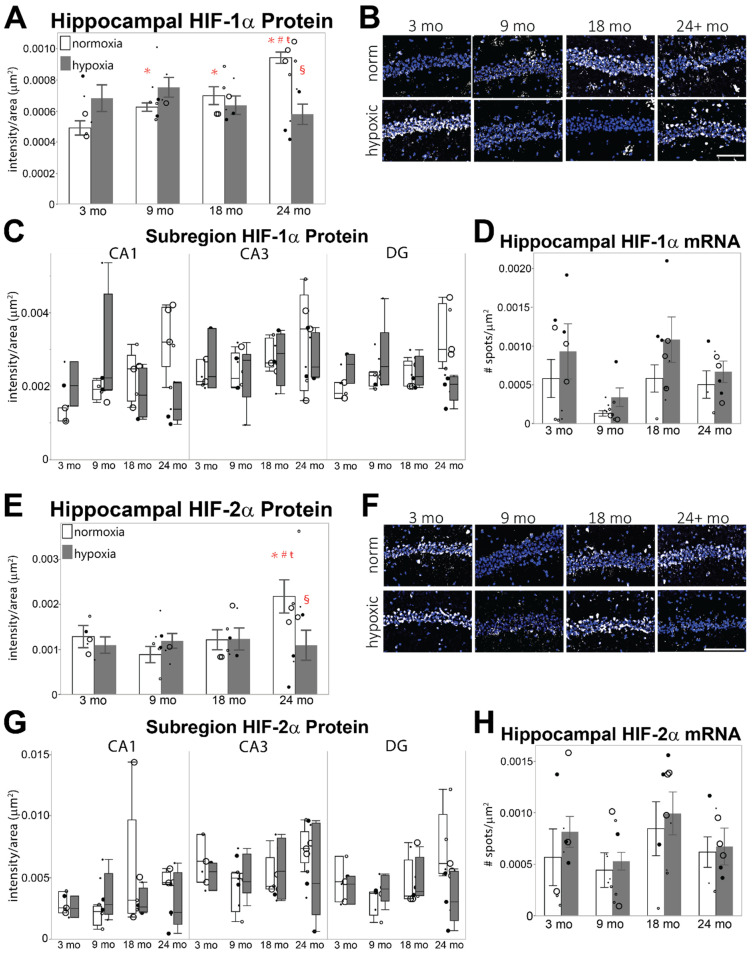
Hippocampal HIF-α protein levels associated with hypoxia. Comparison of HIF-1α and HIF-2α levels in the hippocampus of male (large dot) and female (small dot) mice exposed to room air (normoxic; open circles) or 3% oxygen for 3 h (hypoxia; filled circles). (**A**,**E**) HIF-1α (**A**) and HIF-2α (**E**) mean intensity/μm^2^ in total hippocampus of different aged mice exposed to room air or hypoxia. (**B**,**F**) Representative images of HIF-1α (**B**) or HIF-2α (**F**) (illustrated in white) merged with DAPI (blue) in the hippocampus of different aged mice exposed to room air or hypoxia. (**C**,**G**) HIF-1α (**C**) or HIF-2α (**G**) protein measured as intensity/μm^2^ within hippocampal subregions (CA3, CA1, and dentate gyrus [DG]) of different aged mice exposed to room air or hypoxia. (**D**,**H**) mRNA levels of HIF-1α (**D**) or HIF-2α (**H**) measured by fISH in sections from the same aged mice exposed to room air or hypoxia. Results are reported as the number of spots counted per μm^2^. Scale bar = 150 μm; *p* ≤ 0.05; * = compared to 3 mo normoxic control; # = compared to 9 mo; ŧ = compared to 18 mo; § = interaction between age and hypoxia.

A significant interaction between aging and hypoxia was observed in the hippocampus of 24-month-old mice when compared to 3-month-old mice (F = 5.2533, *p* ≤ 0.05; Figure 4A,B). Following acute hypoxia, the mean intensity of HIF-1α increases in the hippocampus of 3-month-old mice. However, the HIF-1α response to hypoxia is blunted in 9 mo and 18 mo hippocampi, with no significant differences from mice exposed to normoxia, and is blunted in the hippocampus of 24-month-old mice exposed to hypoxia. The hypoxic response observed in 3 mo mice appears to occur mainly in the CA1 subregion and, to a lesser extent, in the DG (Figure 4C). The muted response observed in the 24 mo mouse is also restricted to the CA1 and DG subregions. There is also a trend for differences in hippocampal HIF-1α mRNA expression in the hippocampus (F = 2.1944, *p* = 0.0614; Figure 4D), in which HIF-1α transcription is depressed in 9 mo mouse hippocampi, but overall expression of HIF-1α mRNA increases under hypoxia.

Although there were no significant increases in mean HIF-2α intensity following acute hypoxia in the 3 mo, 9 mo, and 18 mo hippocampi, there is significantly lower HIF-2α intensity in the hippocampi of 24 mo mice following hypoxia (F = 2.2927, *p* = 0.05; Figure 4E,F). The difference is most pronounced within the DG (Figure 4G). No significant differences in Epas1 mRNA expression were observed at any age (Figure 4H).

### 3.3. HIF-α Controlled Transcription Is Altered in Aged Hippocampus at Baseline

**Figure 5:**
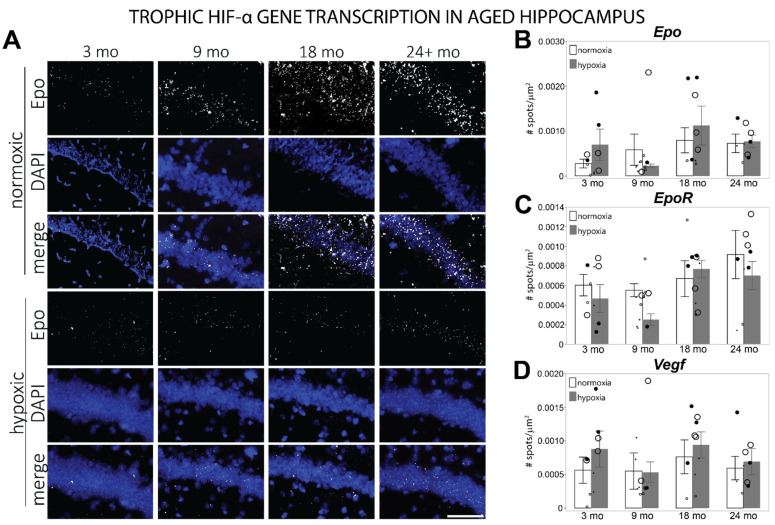
mRNA levels of trophic genes transcribed by HIF-α in the hippocampus of different aged mice exposed to room air or hypoxia. (**A**) Representative images of *Epo* mRNA labeled by fISH in the CA1 region of the hippocampus. (**B**) *Epo* mRNA levels counted in the hippocampus of 3 mo, 9 mo, 18 mo, and 24 mo mice exposed to room air or hypoxia. (**C**) *EpoR* mRNA levels counted in the hippocampus of 3 mo, 9 mo, 18 mo, and 24 mo mice exposed to room air or hypoxia. (**D**) *Vegf* mRNA levels counted in the hippocampus of 3 mo, 9 mo, 18 mo, and 24 mo mice exposed to room air or hypoxia. Results are reported as the mean number of spots counted per area (μm^2^). Scale bar = 100 um; small dots = female mice; large dots = male mice; normoxic = open circles); 3% oxygen for 3 h = filled circles.

To investigate if gene transcription by HIF-α changes during aging, dorsal hippocampal slices were probed using fISH for mRNA representing vital subsets of cellular activity that is controlled by HIF-1α during hypoxia. One of the best-documented effects of HIF-α is in controlling the transcription of *Epo* and *Vegfa*, which are involved in trophic signaling to improve vascularization and red blood cell development. In the hippocampus, EPO also serves as a neuromodulator [45,54,58,59,60], modulating neuronal signaling through the activation of its receptor, EpoR [61]. Although it appears that there is a trend for *Epo* mRNA to be elevated three-fold (Figure 5A,B) and for *EpoR* mRNA to increase by about 33% in aged hippocampus (Figure 5C, Supplemental Figure S1a), these differences were not significantly different from 3 mo, possibly due to the increased individual variability which occurs in aging [62,63]. No differences in basal *Vegfa* mRNA levels were observed (Figure 5D, Supplemental Figure S1b). No differences due to hypoxia were observed in *Epo* (F = 1.0647, *p* = 0.4081), *Vegfa* (F= 0.4979, *p* = 0.829), or *epoR* (F = 2.1493, *p* = 0.0664) transcription in any age group.

**Figure 6:**
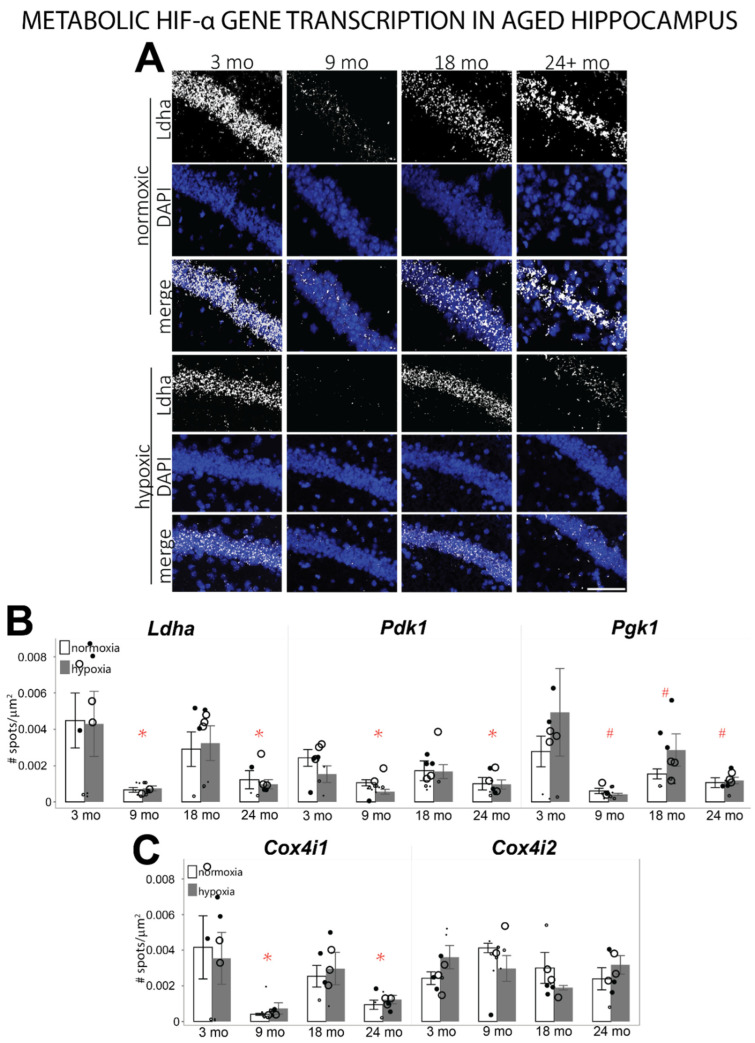
mRNA levels of metabolic genes transcribed by HIF-α in the hippocampus of different aged mice exposed to room air or hypoxia. (**A**) Representative images of *Ldha* mRNA labeled by fISH in the CA1 region of the hippocampus. (**B**) *Ldha, Pgk1,* and *Pdk1* mRNA levels counted in the hippocampus of 3 mo, 9 mo, 18 mo, and 24 mo mice exposed to room air or hypoxia. (**C**) *Cox4i1* and *Cox4i2* mRNA levels counted in the hippocampus of 3 mo, 9 mo, 18 mo, and 24 mo mice exposed to room air or hypoxia. Results are reported as the mean number of spots counted per area (μm^2^). Scale bar = 100 um; *p* ≤ 0.05; * = compared to 3-mo normoxic control; # = interaction with hypoxia; small dots = female mice; large dots = male mice; normoxic = open circles); 3% oxygen for 3 h = filled circles.

Another major HIF-α controlled pathway is the metabolic switch from oxidative phosphorylation to glycolysis during hypoxia (Figure 1). Phosphoglycerate kinase 1 (*Pgk1*) and pyruvate dehydrogenase kinase 1 (*Pdk1*) facilitate the conversion of glucose to pyruvate, while lactose dehydrogenase A (*Ldha*) facilitates the formation of lactate from pyruvate, preventing the transportation of pyruvate into the mitochondria. HIF-α modulates mitochondrial energetics by preferentially transcribing cytochrome c oxidase-4-isoform-2 (*Cox4i2*) over isoform-1 (*Cox4i1*). *Cox4i2* has a lower affinity for oxygen, reducing mitochondrial oxygen usage [62]. A main effect of age was observed in the transcription of most of the metabolic genes investigated. The transcription of *Ldha* (F = 4.1966, *p* ≤ 0.05), *Pdk1* (F = 2.7177, *p* < 0.08), and *Cox4i1* (F = 4.2433, *p* ≤ 0.05) was significantly decreased in 9-month- and 24-month-old hippocampi compared to 3-month-old hippocampus (Figure 6A–C, Supplemental Figures S2a and S3a). *Pgk1* mRNA was significantly decreased in all age groups compared to 3 mo hippocampus (F = 5.1628, *p* ≤ 0.05; Figure 6B, Supplemental Figure S2b). A non-significant increase in *Cox4i2* mRNA was observed in 9 mo hippocampus (Figure 6C, Supplemental Figure S3b). No differences due to hypoxia were observed in any age group for this subset of genes. 

**Figure 7:**
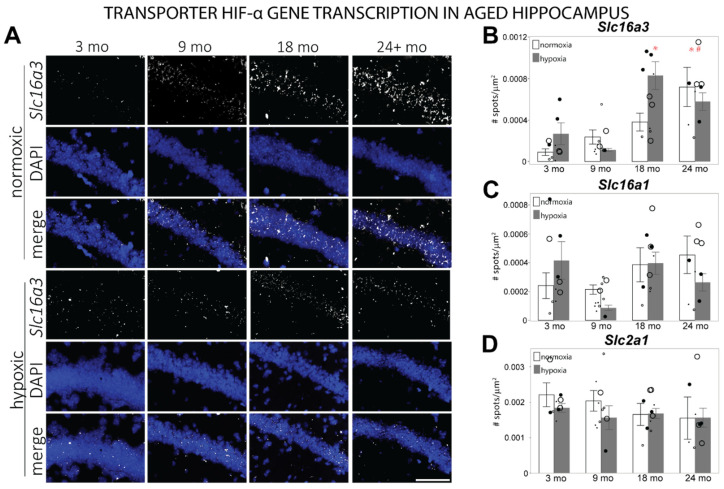
mRNA levels of transporter genes transcribed by HIF-α in the hippocampus of different aged mice exposed to room air or hypoxia. (**A**) Representative images of *Slc16a3* mRNA labeled by fISH in the CA1 region of the hippocampus. (**B**) *Slc16a3* mRNA levels counted in the hippocampus of 3 mo, 9 mo, 18 mo, and 24 mo mice exposed to room air or hypoxia. (**C**) *Slc16a1* mRNA levels counted in the hippocampus of 3 mo, 9 mo, 18 mo, and 24 mo mice exposed to room air or hypoxia. (**D**) *Slc2a1* mRNA levels counted in the hippocampus of 3 mo, 9 mo, 18 mo, and 24 mo mice exposed to room air or hypoxia. Results are reported as the mean number of spots counted per area (μm^2^). Scale bar = 100 um; *p* ≤ 0.05; * = compared to 3-mo normoxic control; # = interaction with hypoxia; small dots = female mice; large dots = male mice; normoxic = open circles); 3% oxygen for 3 h = filled circles.

HIF-α also controls the transport of glucose and lactose across cellular membranes through the genetic transcription of glucose transporter 1 (*Slc2a1*) and monocarboxylate transporters 1 and 4 (*Slc16a1* and *Slc16a3*), respectively. fISH reveals a main effect of age in hippocampal *Slc16a3*, with higher transcription in 18 mo and 24 mo hippocampi as compared to 3 mo (F = 6.9085, *p* ≤ 0.05, Figure 7A,B). A significant interaction between age and hypoxia is observed in the 24 mo group compared to 3 mo hippocampus, in which the transcription of *Slc16a3* under hypoxia is suppressed in 24 mo hippocampus. *Slc16a1* mRNA transcription exhibits no differences due to aging (Figure 7C, Supplemental Figure S4a). Alternatively, *Slc2a1* mRNA exhibits a non-significant trend of decreased expression in aging hippocampus (Figure 7D, Supplemental Figure S4b). No significant differences in *Slc2a1* or *Slc16a1* transcription under hypoxia were observed at any age (*Slc2a1*: F = 0.6024, *p* = 0.7492; *Slc16a1*: F = 2.0442, *p* = 0.0797; Figure 7C,D).

## 4. Discussion

This study is the first to fully characterize HIF-α isoforms and their associated gene products within the hippocampi of adult-aged mice. Most previous investigations into cerebral expression and action of HIF-α during aging have utilized rats as the animal model and used whole brain or cortical homogenates. These findings will facilitate investigations to define altered mechanisms of HIF-α regulation and activity with aging and how those changes impact hippocampal-dependent memory using commercially available transgenic mouse models.

Basal protein expression of HIF-1α and HIF-2α accumulates in the aged mouse hippocampus, as illustrated by IHC (Figure 3), and the accumulation of hippocampal HIF-1α protein under hypoxia declines with age. Based on previous studies of HIF expression under acute hypoxia, this study used 3 h of 8% O_2_ to investigate the effect of age on hypoxic stabilization and the activation of HIF-α transcription. As this has not been performed in aged mouse hippocampal tissue before, it is possible that peak HIF-α expression under hypoxia occurs at a different time point, but these results demonstrate that there are differences in aged hippocampus which may influence functional outcomes. The transcription of *Cox4i1*, the predominate form of cytochrome c oxidase during oxidative phosphorylation, is suppressed with age (Figure 6E), suggesting that mitochondrial efficiency may be altered in aged hippocampus, although we do not observe an age-related increase in the HIF-1α induced *Cox4i2* isoform. Simultaneously, elevated transcription of *Slc16a3*, which codes the lactate transporter MCT4, and suppressed transcription of *Slc2a1*, the glucose transporter GLUT1, indicate an increased reliance upon lactate in aged hippocampus (Figure 7). However, transcription of genes which facilitate lactate production, *Ldha*, *Pdk1*, and *Pgk1*, are suppressed in aged hippocampus (Figure 6A–D). Taken as a whole, these results suggest that mild chronic hypoxia may be present in aged hippocampus, as well as possibly altered gene transcription by HIF-α. On the other hand, these changes may indicate that adaptive homeostatic mechanisms exist in aged hippocampus [32]. Adaptive homeostasis generally declines with age [15], suggesting that it is the former scenario, and one aspect of future studies will be to determine whether HIF-α-induced transcription is indeed impaired in aged hippocampus.

The neuroprotective roles of HIF-1α and HIF-2α are complex and are likely hypoxic paradigm and cell/region-type specific. The inhibition of PHDs in the cortex elevates HIF, reduces apoptosis and reactive oxidative species (ROS) following ischemia, and improves cognitive outcomes [64,65,66]. Similarly, PHD inhibition in hippocampal slices was shown to increase HIF-1α expression and improve the recovery rate of excitatory post-synaptic potentials following a single hypoxic exposure [67]. Loss of HIF-2α in astrocytes reduces fear-conditioned memory retention [34], whereas loss of HIF-1α in neural progenitor cells impairs learning [68]. Other studies have reported that inhibition or knock-out of HIF-α in the brain prevents neural apoptosis following ischemia or intermittent hypoxia [57,69,70]. Under paradigms of intermittent hypoxia, oxidative stress is associated with negative cognitive outcomes [69,71,72]. Recent studies indicate that intermittent hypoxic elevation of reactive oxygen species may be induced by HIF-1α [70,71].

The involvement of HIF-α in hypoxic preconditioning paradigms involves limited acute hypoxic exposures prior to more extreme hypoxia [73,74,75,76,77] and activates gene pathways which improve metabolic efficiency and reduce reactive oxygen species formation under hypoxia [77,78,79,80,81]. Proteins associated with HIF-α, such as EPO, GLUT1, and LDHA, support synaptogenesis and neural connectivity during either hypoxia or aging and improve cognitive outcomes [43,44,45,82,83,84,85,86,87]. Furthermore, lactate-dependent glycolysis is necessary for spatial memory acquisition, suggesting HIF-1α may facilitate synaptic signaling [46]. Confounding the issue is the fact that HIF-1α is not the only transcription factor targeting the glycolysis pathway; therefore, redundant transcription pathways may be compensatory throughout aging [88]. In this study, the transcription of metabolic genes associated with HIF-1α does not reflect observed changes in HIF-1α protein with age. This may be due to impairment in HIF-1α transcription during aging, but it may also reflect the action of other transcription factors. Therefore, it will be necessary to establish the mechanisms by which HIF-α may support synaptic plasticity in the hippocampus and if these mechanisms change during aging.

Adaptive homeostasis, including the HIF-α pathway, declines with age across species [15,89]. Furthermore, aging is associated with slower hippocampal-dependent memory acquisition [89,90,91,92]. Although age itself is not associated with neuronal loss within the hippocampus [92], heart failure, anemia, cerebral blood flow alternations, obstructive sleep apnea, ischemia, and TBI are all associated with hypoxia, damage to the hippocampus, and impaired cognitive function [13,71,93,94,95]. Hypoxia normally induces an increase in micro-vessel density [96] in the CA1 and CA3 regions of the hippocampus, which is lost with age [54,60,97], suggesting that the reduction in HIF-α protein during hypoxia observed in this study may set the stage for aging-related cognitive decline associated with hypoxic disease onset. 

The age-related increase in HIF-α proteins within the CA1 and DG subregions of the hippocampus (Figure 3) may serve to modulate energy needed to maintain basal cognition, as these regions receive synaptic inputs necessary for memory acquisition and consolidation. Aging is associated with lower post-synaptic density (PSD) in the CA1 and reduced perforated synapses in the DG [92]. Long-term potentiation (LTP) in CA1 synapses models changes in synaptic strength, observed during learning. In aged rodents, the incidence of LTP decreases in the Schaffer collaterals of the CA1 (Figure 2), requires a higher stimulus to initiate LTP, and is associated with reduced outcomes in hippocampal-dependent tasks [91]. Gozal et al. [13] reported that intermittent hypoxia acutely induces apoptosis in the CA1 subregion and disrupts the architecture of neuronal excitatory N-methyl-D-aspartate receptors (NMDAR). Patients with heart failure exhibit hippocampal volume loss, primarily within the CA1 but also in the CA3 and subiculum subregions, suggesting that they have an elevated risk of cognitive decline [94]. Deleting NMDARs in the CA1 abolishes LTP and impairs spatial memory in young adult mice [98]. Intermittent hypoxia reduces excitatory post-synaptic potentials in the CA1 region [71,99], likely through attenuated NMDAR expression [71], while simultaneously elevating HIF-α and NADPH oxidase. NADPH oxidase increases oxidative stress and is implicated in cognitive impairment associated with neurodegeneration, ischemia, and traumatic brain injury [80]. Future studies will investigate whether age-related elevation of basal HIF-α contributes to functional cognitive outcomes by decreasing NMDAR expression and/or increasing oxidative stress within the CA1.

Although other groups have reported that HIF-1α decreases and HIF-2α increases in aged cortical structures and that they do not respond to hypoxia [53,55,56], our results are in agreement with the few studies which have focused specifically on HIF-1α expression within the hippocampus using IHC and which report elevated HIF-1α with age [56,100]. Further, we also do not observe a hypoxic response of either HIF-α isoform in the aged groups (Figure 3). Reduced perfusion [19] and neurovascular coupling in aged hippocampus [97,101] likely interacts with high synaptic oxygen demand in the hippocampus to set the stage for mild chronic hypoxia to manifest throughout aging. These findings are in accordance with studies demonstrating that mitochondrial dysfunction, reduced hemodynamic responses, and neurovascular coupling are all implicated in age-related neurodegenerative diseases [20,101,102,103,104].

Even more concerning evidence that HIF-α is necessary for memory stems from studies evaluating individuals with Alzheimer’s disease. HIF-1α and EPO receptor proteins are elevated in the hippocampus of patients with Alzheimer’s disease along with a decrease in GLUT1, GLUT3, and EPO protein [83,93]. Thus, age-related increases in hippocampal HIF-1α likely lead to impaired glycolysis, while changes in EPO are associated with altered HIF-2α. In this study, metabolic support of LTP and synaptic plasticity may be diminished due to differential basal HIF-1α in the aged hippocampus (Figure 3 and Figure 6), while attenuated trophic responses which regulate oxygen delivery are affected by HIF-2α. Thus, the role of HIF-α isoforms in hippocampal synaptic plasticity, functional cognitive outcomes during aging, and mechanisms contributing to the basal increase of HIF-α seen in this study are all necessary areas of investigation.

## Data Availability

Data sharing is not applicable to this article.

## Supplementary Materials

The following are available online at https://www.mdpi.com/article/10.3390/cells11030423/s1, Figure S1a: *EpoR*.; Figure S1b: *Vegfa*.; Figure S2a: *Pdk1*.; Figure S2b: *Pgk1*.; Figure S3a: *Cox4i1*.; Figure S3b: *Cox4i2*.; Figure S4a: *Slc16a1*.; Figure S4b: *Slc2a1*.
