# Supplementary material for: Aged Mouse Hippocampus Exhibits Signs of Chronic Hypoxia and an Impaired HIF-Controlled Response to Acute Hypoxic Exposures
Source: Cells. 2022 Jan 26;11(3):423. doi: 10.3390/cells11030423 (PMC8833982; doi:10.3390/cells11030423)

Supplemental Figure S1a: EpoR

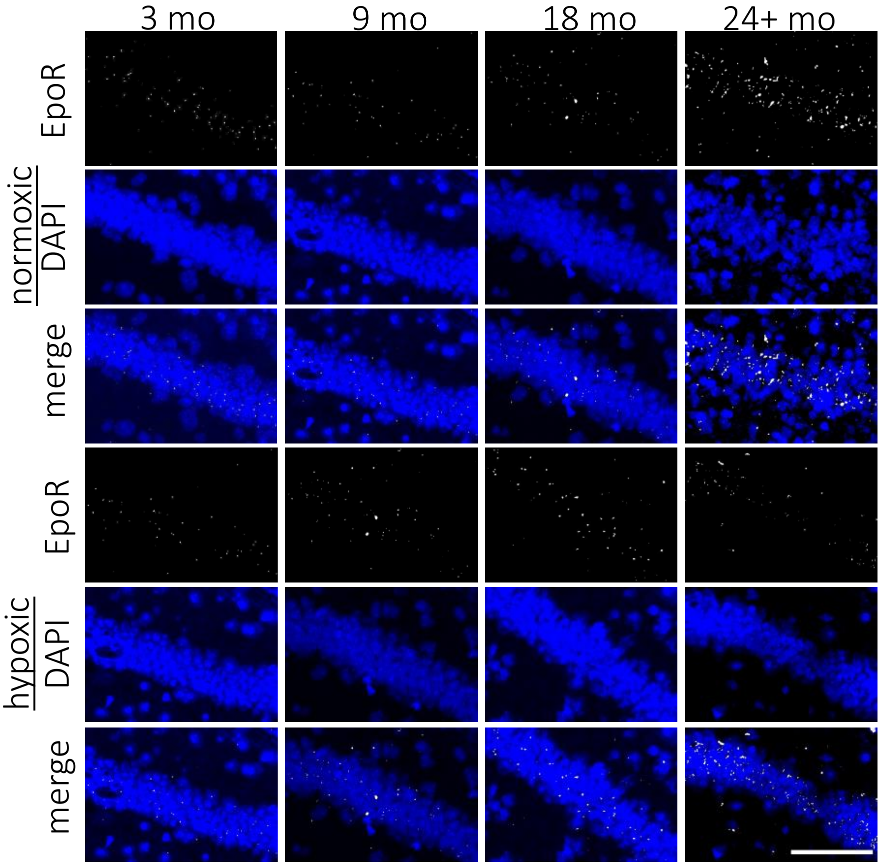

Supplemental Figure S1b: Vegf

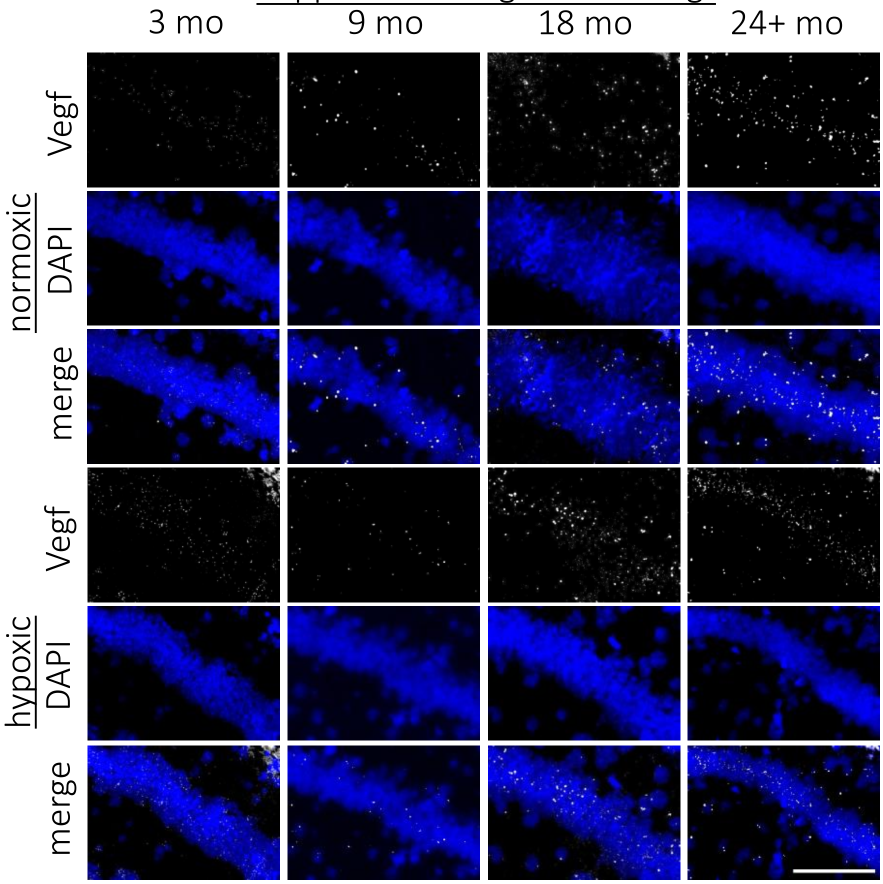

Supplemental Figure S2a: Pdk1

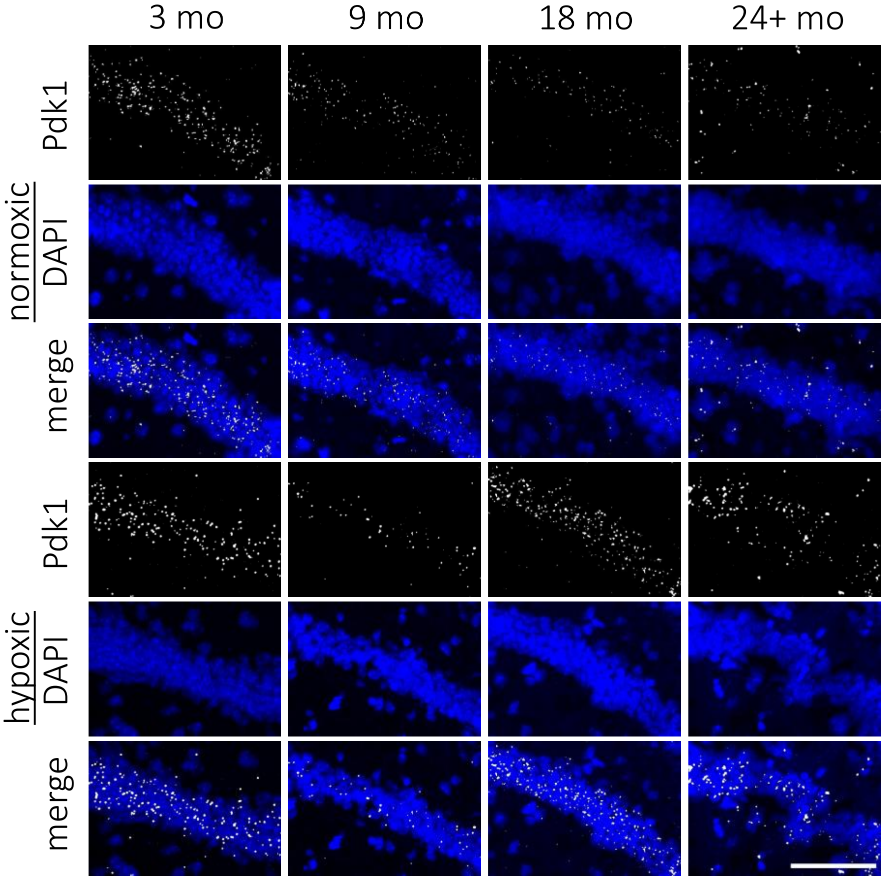

Supplemental Figure S2b: Pgk1

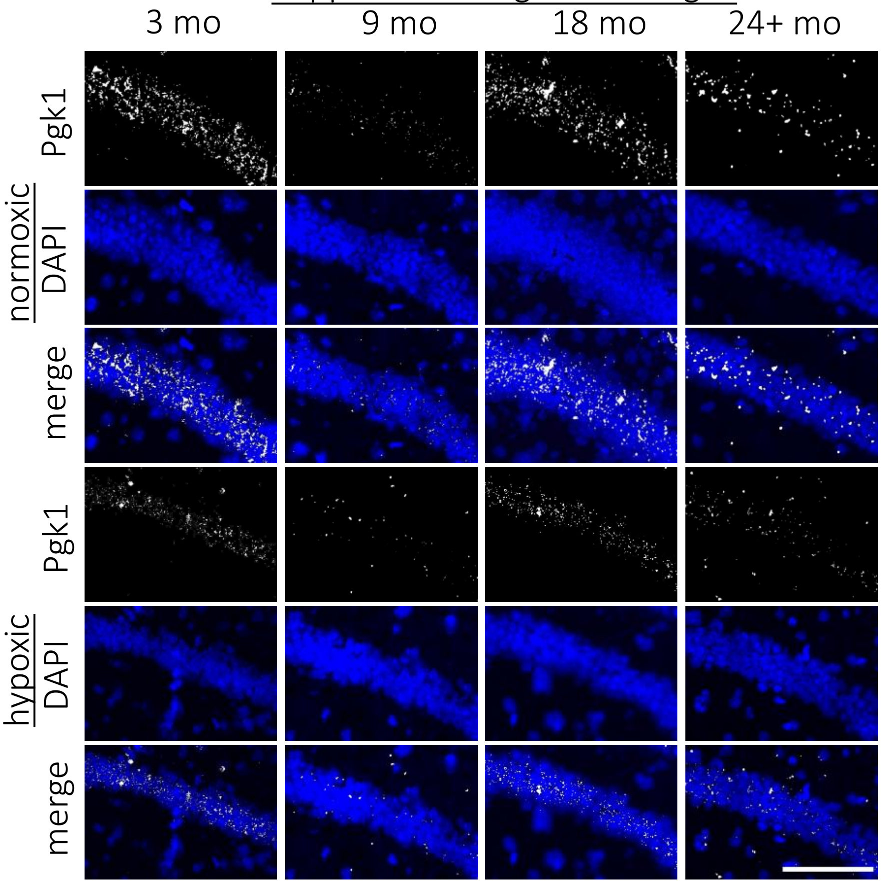

Supplemental Figure S3a: Cox4i1

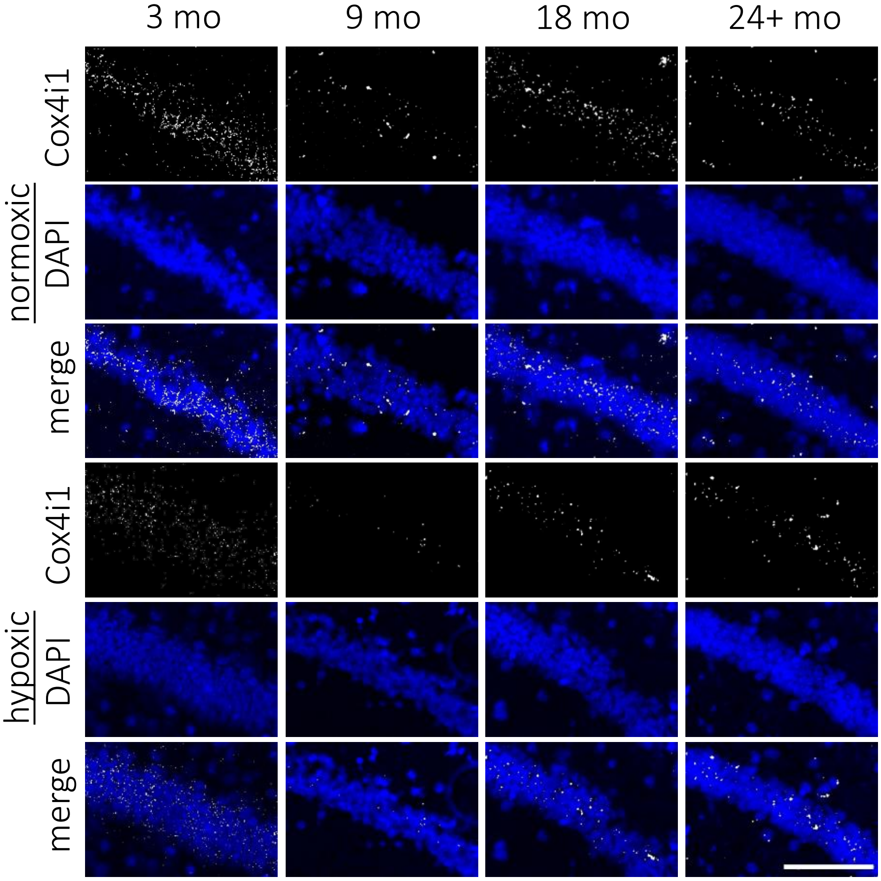

Supplemental Figure S3b: Cox4i2

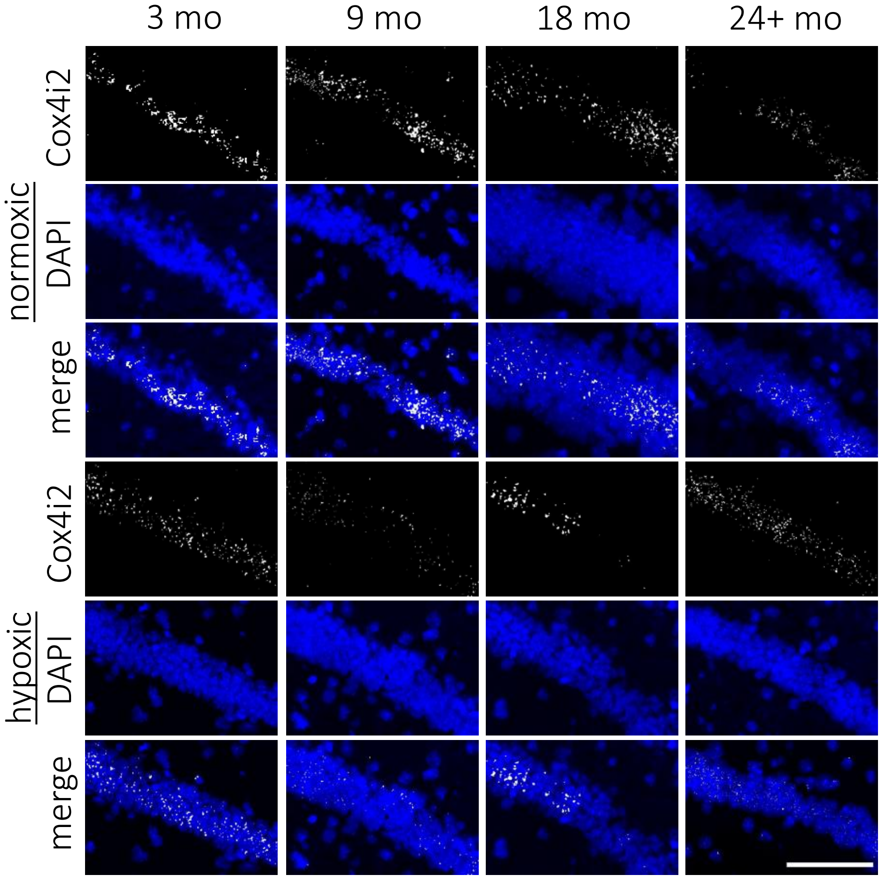

Supplemental Figure S4a: Slc16a1

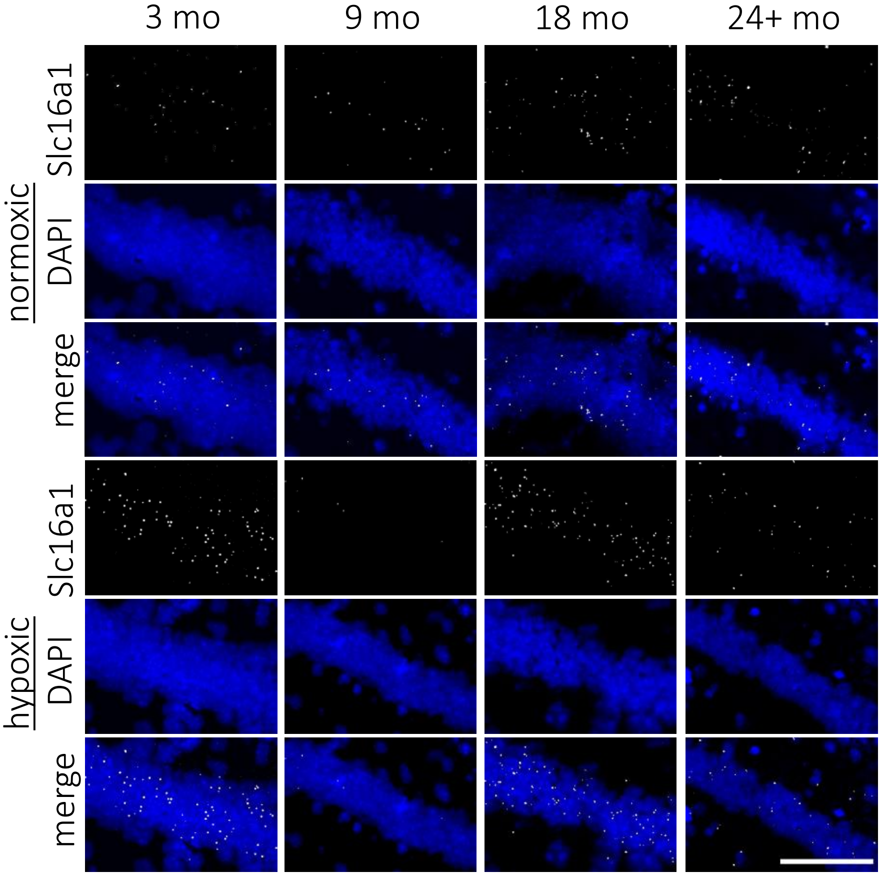

Supplemental Figure S4b: Slc2a1

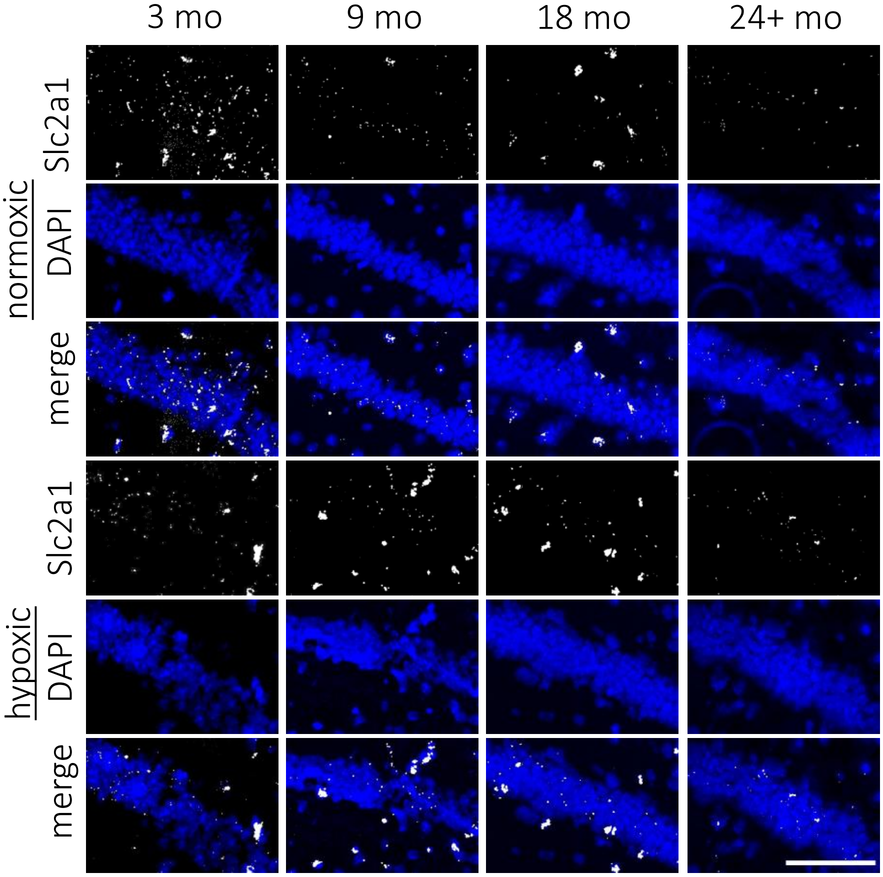

Supplement: Supplementary file 1 [file cells-11-00423-s001.zip › cells-1530887-supplementary.pdf]
